# Effect of Low-intensity Exercise on Physical and Cognitive Health in Older Adults: a Systematic Review

**DOI:** 10.1186/s40798-015-0034-8

**Published:** 2015-10-20

**Authors:** Andy C. Y. Tse, Thomson W. L. Wong, Paul H. Lee

**Affiliations:** 1 Department of Health and Physical Education, Hong Kong Institute of Education, Tai Po, Hong Kong, China; 2 Institute of Human Performance, The University of Hong Kong, Pokfulam, Hong Kong, China; 3 School of Nursing, Hong Kong Polytechnic University, Hunghum, Hong Kong, China

**Keywords:** Low-intensity exercise, Physical health, Cognitive health, Older adults

## Abstract

**Background:**

It is well known that physical exercise is important to promote physical and cognitive health in older population. However, inconsistent research findings were shown regarding exercise intensity, particularly on whether low-intensity exercise (1.5 metabolic equivalent tasks (METs) to 3.0 METs) can improve physical and cognitive health of older adults. This systematic review aimed to fill this research gap. The objective of this study is to conduct a systematic review of the effectiveness of low-intensity exercise interventions on physical and cognitive health of older adults.

**Methods:**

Published research was identified in various databases including CINAHL, MEDLINE, PEDro, PubMed, Science Direct, SPORTDiscus, and Web of Science. Research studies published from January 01, 1994 to February 01, 2015 were selected for examination. Studies were included if they were published in an academic peer-reviewed journal, published in English, conducted as randomized controlled trial (RCT) or quasi-experimental studies with appropriate comparison groups, targeted participants aged 65 or above, and prescribed with low-intensity exercise in at least one study arm. Two reviewers independently extracted the data (study, design, participants, intervention, and results) and assessed the quality of the selected studies. Fifteen studies met the inclusion criteria. Quality index ranged from 15 to 18 mean = 18.3 with a full score of 28, indicating a moderate quality. Most of the outcomes reported in these studied were lower limb muscle strength (*n* = 9), balancing (*n* = 7), flexibility (*n* = 4), and depressive symptoms (*n* = 3).

**Results:**

Out of the 15 selected studies, 11 reported improvement in flexibility, balancing, lower limb muscle strength, or depressive symptoms by low-intensity exercises.

**Conclusions:**

The current literature suggests the effectiveness of low-intensity exercise on improved physical and cognitive health for older adults. It may be a desired intensity level in promoting health among older adults with better compliance, lower risk of injuries, and long-term sustainability.

## Key Points

- Low-intensity exercise offers both physical and cognitive health benefits to older adults.
- Low-intensity exercise is useful to induce health benefits for high-risk population such as physical frail older adults.
- Low-intensity exercise induces better exercise adherence as relative to moderate and high intensity exercise.

## Background

In recent decades, many parts of the world have aging populations, including the UK, Canada, and the USA [1–7]. Hong Kong is no exception. It is estimated that the number of adults aged 65 and older in Hong Kong will increase by 1.6 million to 3.6 million by 2041—with approximately one in three persons being older adults, up from the current proportion of one in seven [8]. With advancing age and declining functional capacity [9], older adults are more prone to health-related problems such as declining muscular strength and cardiovascular endurance [10, 11]. A survey conducted in Hong Kong revealed that 70.4 % of older adults reported to suffer from at least one chronic disease [12], which describes a high rate of morbidity and mortality among older adults [13, 14]. Moreover, with declining cognitive functions, risk of dementia and severity of depressive symptoms were unsurprisingly increased [15–21].

With such a dramatic increase in the older adult population, one may foresee that medical costs associated with older adults will inevitably continue to grow. According to the Hong Kong Government, people aged 65 years and over constituted 13.2 % of the whole Hong Kong population but consumed 15.8 % of total government expenditure in the 2013–2014 financial year [22]. This situation presents challenges to various healthcare service providers for older adults. The search for optimal preventive care and public health interventions that promote physical and cognitive health among aging populations is thus crucial for city planners, healthcare professionals, and stakeholders.

Exercise is one such preventive public health intervention. It is widely reported to be effective in reducing all-cause mortality, cardiovascular disease, and cancer [23–28]. The term “exercise” is defined as a regular structured program of physical activity [29, 30], where “physical activity” is defined as an activity in daily life that may be categorized as occupational, sports, conditioning, household, or other [29]. Previous studies have shown that exercise can change postural control functioning in older adults, which leads to a reduced fall risk and better maintenance of upright stances [31–34]. In addition, exercise is associated with cognitive health in older adults by delaying the symptoms of cognitive diseases, such as dementia, and mood disorders, such as depression [35–37].

Although the benefits of exercise have been well documented in the literature, there is a lack of universal agreement on the frequency, intensity, and types of exercise required for health promotion among older adults. “Exercise intensity” is defined as how hard the exertion is during exercise [38] and is typically measured in metabolic equivalent task (MET) [39]. One MET is defined as the rate of energy expenditure at rest [40]. Activities with METs between 3.0 and 6.0 are considered to have moderate intensity, whereas exercise intensities above 1.5 METs and below 3.0 METs are considered to be low [38, 40, 41]. Typical low-intensity exercises for older adults include light walking, stretching, lifting hand weights, sit-ups, and push-ups against the wall [42]. Combination exercises with low intensity are often administered as exercise programs for older adults [43]. Currently, considerable research has shown that activities with at least moderate intensity (including running, tennis, and aerobics) could lower the risk of all-cause mortality, type 2 diabetes, hypertension, stroke, colon cancer, breast cancer, depressive symptoms, and dementia [44–48]. In contrast, the effects of low-intensity exercise—both lower in injury risk and generally more affordable to older adults—did not receive the same attention as did those of moderate intensity exercises. Previous literature on the effectiveness of low-intensity exercise in older adults has shown conflicting evidence. One study showed that low-intensity exercise was not associated with health improvements [49]; however, others have demonstrated significant improvement in health [50, 51]. Consequently, it remains debatable whether low-intensity exercise would be effective in improving physical and cognitive health in older adults. The purpose of the present study was to conduct a systematic review to draw a conclusion about the effectiveness of low-intensity exercise in older adults.

## Methods

### Information Sources

This review adhered to the Preferred Reporting Items for Systematic Reviews and Meta-Analysis (PRISMA) guidelines [52]. Six electronic databases (CINAHL, MEDLINE/ PubMed, PEDro, Science Direct, SPORTDiscus, and Web of Science) were used to access as many relevant articles as possible.

### Search Strategy and Data Items

A systematic search strategy was conducted using the electronic databases with varying combinations of the following terms found in the title, abstract, or keyword fields: “exercise OR low intensity exercise,” “health OR physical exercise OR cognitive exercise,” and “older adult OR elderly.” For example, “exercise OR ‘low intensity exercise’ AND health AND ‘older adult’” was searched in the CINAHL electronic database, and 218 relevant studies were found. Searches included papers published from 1994 to March 01, 2015.

### Eligibility Criteria and Study Selection

To be included in this systematic review, articles were required to be as follows: published in an academic, peer-reviewed journal; published in English; conducted as a randomized controlled trial (RCT) or as a quasi-experimental study with appropriate comparison groups; targeted to participants aged 65 or older; and inclusive of an exercise intervention of differing intensity levels, with low-intensity exercise in at least 1 study arm. The low-intensity exercise referred to any exercise level greater than 1.5 METs but less than 3 METs [38, 40]. There was no limit imposed on the duration, frequency, or type of exercise intervention, and no specific physical or cognitive outcome measures were stipulated.

### Data Extraction and Quality Assessment

The included studies were analyzed and cross-checked independently by two authors to extract the following information: study design, objectives and discussion, participants and eligibility criteria, intervention used, variables and measurement, key outcomes, and conclusions. A data extraction form was used to standardize the data extraction process. Discussion was conducted in cases of disagreement, and a consensus would be reached on whether the studies in question would be included in the present review.

The two reviewers independently assessed the quality of the articles with the use of the quality index (QI) [53] shown in Appendix 1. The quality index is a well-established quality assessment tool for systematic reviews and social care interventions. It comprises 27 items that are categorized into five subscales: reporting (10 items), external validity (3 items), internal validity-bias (7 items), internal validity-confounding (6 items), and power (1 item) [53]. All item answers are indicated as “yes” (score = 1), “no” (score = 0), or “unable to determine” (score = U). A higher score represents higher quality. In cases of disagreement in this review, a third reviewer was consulted to resolve any discrepancies.

### Data Synthesis

The selected studies were divided into two domains—physical health and cognitive health. Within each domain, the low-intensity exercise group was compared either with a control or another exercise group.

## Results

### Search Results

**Fig. 1:**
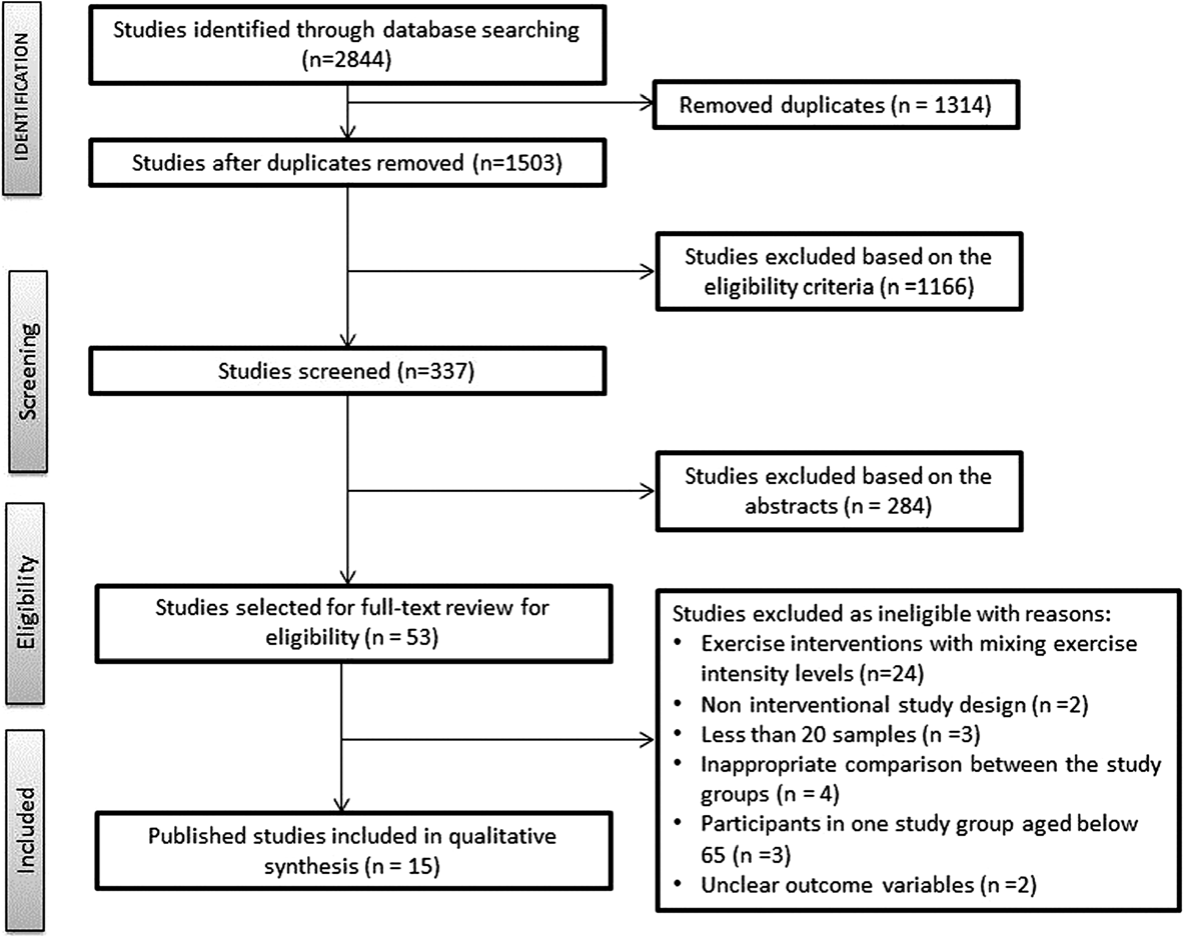
Flowchart describing the selection process of the included studies

A total of 2884 relevant published studies were initially found from searches, as shown in Fig. 1. After initial screening of abstracts against the inclusion criteria, 337 studies remained. After final analysis, 15 of those studies [54–68] met the inclusion criteria, with articles from CINAHL (*n* = 2), MEDLINE/PubMed (*n* = 2; included studies from MEDLINE and PubMed were identical), PEDro (*n* = 7), Science Direct (*n* = 2), SPORTDiscus (*n* = 1), and Web of Science (*n* = 1).

### Quality Assessment

Quality index scores ranged from 15 to 20 (mean = 18.3), with the highest possible score being 28. Most studies (*n* = 12) scored 18 or higher [54–56, 59–63, 65–68], whereas the rest (*n* = 3) had QI scores of 15 [64], 16 [57], and 17 [58] (Table 1). Inter-rater reliability of both raters was assessed by comparing the total rated scores with the means of the Spearman correlation coefficients and the level of agreement with the κ statistic. Inter-rater reliability was moderate (*ϒ* = 0.61; *κ* = 0.57) [53]. As shown in Table 1, most studies were very clear on reporting and satisfied the criteria of external validity. The most diversifying issues were related to internal validity-confounding criteria such as trials not being blinded or open-labeled, inadequate adjustment for confounding in estimating the effect of low-intensity exercise, and lack of reporting on participant attrition.

**Table 1:** Quality index assessment scale ratings

Study	Reporting	External validity	Internal validity—bias	Internal validity—confounding	Power	QI score
	Full score, 11	Full score, 3	Full score, 7	Full score, 6	Full score, 1	Full score, 28
Blair et al., 2014 [54]	7	2	5	4	0	18
Brown et al., 2000 [55]	8	3	5	3	0	19
Dawe et al., 1995 [56]	7	3	5	4	0	19
DeVito et al., 2003 [57]	6	3	5	2	0	16
Lam et al., 2011 [58]	8	2	4	3	0	17
Li et al., 2005 [59]	8	3	5	2	0	18
Mangione et al., 1999 [60]	8	3	5	3	0	19
Means et al., 1996 [61]	9	3	4	2	0	18
Morey et al., 2009 [62]	7	2	6	3	0	18
Morgan et al., 2004 [63]	8	3	5	3	0	19
Motl et al., 2005 [64]	6	3	4	2	0	15
Rosie et al., 2007 [65]	9	3	5	2	0	19
Schnelle et al., 2003 [66]	8	3	6	3	0	20
Singh et al., 2005 [67]	8	3	6	3	0	20
Wolfson et al., 1996 [68]	9	3	5	3	0	20

### Study Characteristics

All included studies (*n* = 15) were pre- and posttest designs, 10 of which [55, 58, 59, 62, 63, 65–68] were RCTs, and 4 of which [54, 59, 64, 68] included follow-up assessments after the intervention had been completed (Table 2). Sample sizes varied from 20 [55] to 641 [61]. Most of the studies (*n* = 12) were related to the physical health of older adults, and 4 were related to cognitive health (Table 2). All studies used low-intensity exercise of differing types as the study intervention, including low-intensity stationary cycling [59], stretching [56, 59, 61, 64], walking [56, 61, 63], Tai Chi [58, 59, 68], balance training [63, 68], resistance training [64, 67, 68], seated exercise [56, 63], and functional exercise programs [55, 57, 62, 66]. Control groups engaging in their usual physical activities were used in 6 studies [57, 58, 63, 66–68], and 1 study used a viewing of a 15-min exercise program video [58]. In the remaining studies, the intervention groups were compared with other exercise groups such as stretching and toning groups [61], non-obstacle practice groups [63], high-intensity resistance training groups [69], home-based exercise groups [57], delayed intervention groups [56, 64], high-intensity cycling groups [62], and knee extension groups [67]. The duration and frequency of the low-intensity exercise intervention were diverse, ranging from 1 h to 1 year and from one to three times per week.

**Table 2:** Description of studies included in the review

Study	Domain	Design	Sample	Intervention	Key outcomes	Results
Dawe et al., 1995 [56]	Cognitive health	Pre-post test	*N* = 20	Duration, 1 h	Blood pressure = mmHg	Intervention group = significantly increased pulse rate (from 69.2 to 71.2 beats/min) and blood pressure (from 140/75 to 145/73 mmHg) (*p’s* < 0.001)
			Nursing home residents	Intervention group = received the Canadian Red Cross Society’s Senior’s Fun and Fitness program.	Pulse = beats/min	
			Cognitively unimpaired		Three cognitive tests:	
			Male, 4; Female, 16		1. Set test = number of words correctly recalled;	Control group = no differences in pulse rate (between 74.1 to 74.7 beats/min), blood pressure (from 137/74 to 136/72 mmHg), and three cognitive tests (*p’s >* 0.05).
			Intervention group (*n* = 10)	Control group = viewed a 15-min video of low-intensity exercise program		
			Mean age: 83.9		2. Word fluency test = number of words correctly recalled;	
			Control group (*n* = 10)			Between groups, intervention group = showed a better cognitive performance (i.e., improved recall ability in the Set test (40 words to 46 words) than the control group (43 words to 44 words) (*p* < 0.05))
			Mean age = 85.1	Overall intervention compliance = no information	3. Symbol digit test = number of corrected digit encoding	
Means et al., 1996 [61]	Physical health	Pre-post test	*N* = 65	Duration, 6 weeks	Performance score (0 = poorest performance to 3 = best performance; total point = 36) and completion time (in seconds) on a functionally oriented obstacle course	Practice group = significantly decreased the completion time (440.9 to 351.6 s); increased in performance score (24.6 to 26.4) (*p’s* < 0.05)
			Community-dwelling;	Balance and mobility exercise protocol:		
			One or more falls within a year prior to the study	Active stretching, postural control, endurance walking, repetitive muscle coordination exercises		
						Non-practice group = significantly decreased the completion time (319.1 to 293.5 secs); increased in performance score (25.7 to 26.7) (*p’s* < 0.05)
			Gender: no specified		Self-reported falls = number of falls	
				Cognitive intervention sessions	Fall related injuries	Between groups = no significant differences in the completion time performance score (*p >* 0.05)
			Intervention group (*n* = 31)	Practice group = received extra training on an obstacle course along with the exercise protocol		
			Mean age: 75			Between groups = no significant difference in number of falls (*p >* 0.05)
			Control group (*n* = 34)			
			Mean age, 75	Non-practice group = receive no training on an obstacle course but only the exercise protocol		Overall, all participants = decreased the completion time (378 to 321 secs); improved in mean performance scores (from 25.2 to 26.5 points) after the exercise protocols
				Overall intervention compliance = no information		
Wolfson et al., 1996 [68]	Physical health	RCT	*N* = 110	Phase 1	Loss of balance during sensory organization test (LOB) = number of times that participants received support from the experimenter	Balance group = significant improvements in LOB (3.6 to 1.4), FBOS (0.44 to 0.52 % of foot length), SST (12.2 to 16.6 s) (*p’s* < 0.001); no improvement in ISOK (8.0 to 8.1 Nm/kg) and UGV (1.14 to 1.18 m/s) (*p’s >* 0.05)
			Community-dwelling	3-month balance and strength training were provided to the respective groups (45-min per week)		
			Healthy			
			Male, 64; Female, 46			
			Balance group (*n* = 28)	Control group was encouraged to continue their usual activities	Functional Base of Support (FBOS) = % of foot length	Strength group = significant improvements in ISOK (6.5 to 8.0 Nm/kg) and LOB (3.7 to 2.1) (*p’s* < 0.001); no improvements in other measures: FBOS (0.38 to 0.39 % of foot length), and SST (9.1 to 10.0 secs), and UGV (1.08 to 1.17 m/s) (*p’s >* 0.05)
			Mean age, 78.9			
			Strength group (*n* = 28)	Phase 2	Single Stance Time (SST) = seconds	
					Isokinetic strength (ISOK) = Nm/kg	
			Mean age, 80.0	6-month low-intensity balance and strength maintenance programe (Tai Chi training with self-administered home practice) (1 hour per week)	Usual Gait Velocity (UGV) = m/s	Balance and strength group = significant improvements in LOB (3.6 to 1.9), FBOS (0.4 to 0.5 % of foot length), SST (5.4 to 15.1 secs), ISOK (6.8 to 8.0 Nm/kg) (*p’s* < 0.001); no significant improvement in UGV (1.12 to 1.09 m/s) (*p’s >* 0.05)
			Balance and strength group (*n* = 27)			
			Mean age, 79.7			
			Control group (*n* = 27)			
			Mean age, 80.6	Overall intervention compliance = 72 %		Overall, no group differences (*p’s >* 0.10)
Mangione et al., 1999 [60]	Physical health	Pre-post test	*N* = 39	Duration, 10 weeks	Timed chair rise = second	High intensity group = significantly reduced the chair rise time (23.5 to 19.3 secs) and AIMS2 pain score (4.3 to 3.0); significantly increased in 6-min walk (488.0 to 540.6 m), aerobic capacity (11.0 to 13.3 min), and peak oxygen
				Exercise training = Stationary cycling; 1 hour each session; cycle 25 min; 3 times per week	6-min walk test = m	
					Arthritis Impact Measurement Scale 2 (AIMS2) pain score	
				High intensity group = stationary cycling at 70 % heart rate reserve		
					Aerobic capacity time for graded exercise test = min	consumption (1454.1 to 1545.3 ml/min) (*p’s* < 0.01)
				Low-intensity group = stationary cycling at 40 % heart rate reserve		
						Low-intensity group = significantly reduced the chair rise time (23.1 to 19.0 secs) and AIMS2 pain score (3.6 to 3.1); significantly increased in 6-min walk (491.1 to 526.9 m), aerobic capacity time (11.1 to 13.0 min), and peak oxygen consumption (1710.2 to 1807.3 ml/min) (*p’s* < 0.01)
					Peak oxygen consumption = ml/min	
				Overall intervention compliance = no information		
			Suffered from knee osteoarthritis			
			Community-dwelling			
			Male, 13; Female, 26			
			High intensity cycling group (*n* = 19) mean age = 71.1			
			Low-intensity cycling group (*n* = 20)			
			Mean age = 71.0			
Study	Domain	Design	Sample	Intervention	Key outcomes	Results
Brown et al., 2000 [55]	Physical health	RCT	*N* = 87	Duration, 3 months	Physical Performance Test (PPT) score	EXER group = significant improvements on the PPT score (29 to 31 points), strength measures (e.g., isometric knee extension: 62 to 65 ft/lb), ranged of motion (e.g., shoulder flexion: 160 to 165 mm), balance measures (e.g., one-limb stand: 4.1 to 7.6 s), and coordination and response time (358 to 377) (*p’s* < 0.05); no significant improvements in gait measures (*p >* 0.05)
			Community-dwelling	Supervised exercise group = low-intensity supervised exercise program (22 exercises; 3 times per week) targeting all muscle groups	Strength measures = ft/lb	
			<32 points on Physical Performance Test (PPT)		Range of motion = mm	
					Balance measures:	
			Male, 20; Female, 28		Obstacle course = second; functional reach = inch; Romberg = second; one-limb stand = second; balance beam = second)	
			Supervised exercise group (EXER) (*n* = 48)	Home-based flexibility activity group = some of the exercises done in the other group and were not supervised.		
						HOME group = no significant improvements on PPT score (29 to 29 points), strength measures (e.g., Isometric knee extension: 56 to 54 ft/lb), balance measures (e.g., one-limb stand, 4.9 to 5.2 secs) and gait measures (*p’s >* 0.05); significant improvements in range of motion (e.g., should flexion, 159 to 161 mm), balance, gait, coordination/response time (351 to 417 msecs) (*p’s* < 0.05)
					Gait measures : gait velocity = m/min; stride length = m ; stance time = second; swing = % of gait cycle; stance = % of gait cycle; double stance = %	
			Mean age, 83	Overall intervention compliance = no information		
			Home-based flexibility activity group (HOME) (*n* = 39)			
					Coordination/response = msec	
			Mena age, 83			
DeVito et al., 2000 [57]	Physical health	Pre-post test	*N* = 105	Duration, 8–10 months	Mobility measures score	Intervention group = significant improvements in all outcomes (e.g. Balance score: 9.6 to 12.8) (*p’s* < 0.001)
			Had a hospital admission lasting 2 days or more or had been on bed rest for 2 days or more within the past 1 month	Intervention group = 24 sessions (45 min) of 3 sets of low-intensity standard exercise modalities (3 times a week) targeting on flexibility, postural stability, balance and gait (e.g., extend leg up then back down, raise up and down on toes then heels, march in place etc.); continue performing exercise until 1 year after the baseline assessment; Individualization of the program according to participant’s ability and progress	Gait score	
					Balance score	Control group = significant improvements in all outcomes (e.g., Balance score: 9.8 to 10.4) (*p’s* < 0.001)
					Muscle strength score	
						Between groups = intervention group has significant greater samples in improving in gait, balance and mobility measures (*p’s* < 0.001). e.g., 35.1 % of intervention group increased in walking ability while 15.9 % of control groups increased in walking ability (*p* < 0.001)
			Male, 47; Female, 58			
			Intervention group (*n* = 60)			
			Mean age, 80			
			Control group (*n* = 45)			
			Mean age, 81			
				Control group = usual activities		
				Overall intervention compliance = 91 %		
Schnelle et al., 2003 [66]	Physical health	RCT	*N* = 190	Duration, 8 months	Medical conditions (dermatological, genitourinary, gastrointestinal, respiratory, endocrine, neurological and cardiovascular systems, falls, and pain, psychiatric and nutritional disturbances) were extracted	Between groups = intervention group has significant smaller number of falls than the control group (*p’* < 0.05); no difference on other medical conditions and cost of treatment (*p’s* > 0.05)
			Living in nursing home	Intervention group = engaged into the low-intensity functional oriented exercise program: Functional Incidental Training (FIT) (5 days a week; every 2 h between 0800 to 1600)		
			Male, 28; Female, 162			
			Intervention group (*n* = 92)			
			Mean age, 87.3		Cost of treatment	
			Control group (*n* = 98)			
			Mean age, 88.6	Control group = received usual care from NH staff; no change in their physical activity or other measures.		
				Overall intervention compliance = 91 %		
Morgan et al., 2004 [63]	Physical health	RCT	*N* = 229	Duration, 8 weeks	Gait and balance (Tinetti’s gait and balance assessment measures)	Exercise group = 28.6 % participants fell; risk of falling decreased with low baseline physical function (*p* < 0.001); increased fall risk with high physical function (*p* < 0.001)
			Had a hospital admission or bed rest for 2 days or more within the previous month	Exercise group = perform chair-sitting exercise and standing balance exercises; 3 times per week.		
						Control group = 30.9 % participants fell
					Self-reported functional status (SF-36) = range, 0-100	
				Control group = continue their usual activities.	Number of fall for 1 year after the assessment	Overall, 29.7 % of the participants reported a fall during study period
				Overall intervention compliance = 70 %		
			Male, 67; Female, 162			
			Exercise group (*n* = 119)			
			Mean age, 81.0			
			Control group (*n* = 110)			
			Mean age, 80.1			

### Physical and Cognitive Health Outcomes

In terms of physical health, improvements were reported in range of motion [57], endurance [61, 62], gait velocity [60, 68], lower limb muscle strength [53–56, 61, 62, 66, 68], overall pain [60], balance [55. 57, 61, 66–68], and peak oxygen consumption [56, 66]. In terms of cognitive health, significant reduction in depression scores [64, 67] and improved cognitive functions [56, 58] were reported. Overall, the key outcomes of the included studies were lower limb muscle strength, flexibility, balance, and depressive symptoms.

## Discussion

This systematic review examined the effect of low-intensity exercise on physical and cognitive health in older adults. The majority of the studies included showed that low-intensity exercise was effective in improving balance and lower limb muscle strength, as was evident in the reduction of fall frequency and fall risk [59, 61, 63, 66, 68].

Falls are a major cause of morbidity and mortality in older adult populations [69–71]. A previous study showed that the fall rate among community-dwelling older adults aged 65 or older was 26.4 %, and the incidence rate of new fallers was 198 per 1000 persons per year [72]. Serious injuries such as bone fractures [72–74] commonly result from falls. Even for those who did not experience any serious injuries after a fall, the resultant functional deterioration [75, 76], self-rated health [77], and fear of falling [78, 79] may lead to impairment of daily living activities, adversely affecting quality of life.

More than half of the included studies (*n* = 10) supported the benefits of low-intensity exercise intervention towards fall prevention in older adults. Brown et al. [55] designed a 3-month program of low-intensity exercises for older adult participants with minor frailty (Table 2). Participants completed a modified physical performance test involving domains of flexibility, balance, body-handling skill, reaction speed, and coordination before and after the program. Results revealed significant improvements in all physical domains by low-intensity exercise [54]. Moreover, increases in sense of enjoyment and self-rated improvement in physical performance were also reported by the participants after low-intensity exercise.

Similar benefits were also evidenced in Morgan et al.’s study [63], where the researchers employed a physical restoration intervention consisting mainly of a series of low-intensity standing and sitting exercises among a group of clinically defined at-risk older adults (i.e., those who had either a hospital admission or bed rest for 2 days or more within the previous month). In the 1-year fall-tracking period after the study, only 29 % of the study participants reported a fall. This study also [63] found that the low-intensity exercise was more effective for the clinically defined at-risk older participants. Benefits among healthy older adults, however, were questionable; the results suggested low-intensity exercise led to an *increased* fall risk among healthy elders [63]. One possible explanation for the increased fall risk is that those with high physical functioning may have a higher threshold (i.e., a muscle strength level at which the exercise program starts to be effective); therefore, the high-functioning older adults did not benefit from the small increase in muscle strength from the exercise program [80]. Another possible explanation may be that fall risk increases simply by increasing amount of activity [80–82].

It is noteworthy that Tai Chi, a traditional Chinese martial art consisting of a series of slow but continuous body movements [83], is widely accepted to be a low-intensity fall prevention exercise for both high- and low-functioning older adults [59, 83–87]. In 1 study included in this review, the effectiveness of a 6-month Tai Chi program and a 6-month stretching program with identical weekly schedules (three times per week) was compared [59]. This study [59] showed that participants in the Tai Chi group reported fewer falls, lower proportions of falls, and fewer injurious falls than those in stretching group after the 6-month exercise intervention period. Moreover, in contrast to the stretching group, the Tai Chi group also showed improvements in all measures of functional balance, physical performance, and reduced fear of falling [59]. This study added new evidence to support reports that showed certain low-intensity exercises may be better than others at sufficient intensity for reducing fall incidence.

Apart from physical health benefits, the present review also found strong evidence to support the cognitive health benefits of low-intensity exercise. Two of the included studies investigated the effect of low-intensity exercise on depressive symptoms [64, 67]. Both studies showed a reduction in depression symptoms after the low-intensity exercise intervention (with 1 study using the Hamilton Rating Scale of Depression and the other study using the 30-item Geriatric Depression Scale) [67]. These findings suggested that exercise of low-intensity levels might be of adequate intensity to prevent depression—a valuable insight for preventing and treating cognitive health problems among older adults. In Hong Kong, the prevalence of depression among older adults was 11.0 % for men and 14.5 % for women [88]. Globally, it is estimated that major depressive disorder would become the second most prevalent disease among elderly people by 2020 [89]. Depression inflicts enormous suffering on individuals, often promoting social isolation, insomnia, and decreased concentration. Serious depression may even lead to recurrent thoughts of death and suicidal ideation. It also poses various challenges to families and to the community. Physical treatment (e.g., pharmacological and electroconvulsive therapy) and psychosocial treatment are long established and traditional treatments for clinical depression [90]. However, some of these treatments also bring unpleasant side effects that interfere with patients’ quality of life and reduce compliance with prescribed drug therapies [91]. Exercise may help alleviate some of these unpleasant side effects.

Numerous studies have shown that exercise has been effective in reducing depression symptoms [92–96], with moderate intensity exercise being the most frequently prescribed exercise intensity in these studies. Blumenthal et al. [92], for example, conducted an exercise intervention with moderate intensity walking (intensity up to 70 to 85 % of maximum heart rate reserve) for the treatment of major depression among older adults. The authors concluded that moderate intensity walking may be a viable treatment for major depression. Beyond moderate intensity exercise alone, however, low-intensity exercise and physical activity, in general, may also be effective means of coping with depression. In Singh et al.’s study [57], 8 weeks of high-intensity progressive resistance training (80 % of one repetition maximum) was observed to be more effective than the low-intensity training (20 % of one repetition maximum) for alleviating depression symptoms; however, low-intensity exercise was reported to significantly reduce the number of depressive symptoms by 29.0 %. Similar results were also displayed in Motl et al.’s study [64], where both low-intensity exercise groups and walking groups displayed a significant reduction in depressive symptoms after the 6-month intervention and in the 12-month and 60-month follow-up periods [64]. These findings indicated that moderate or vigorous intensity levels of physical activity might not be necessary to achieve cognitive health benefits. Implications for healthcare professionals may include increasing prescription of low-intensity exercise among older adults with cognitive health problems. More research should be conducted to confirm the relationship between low-intensity exercise and cognitive health.

This review also evaluated the quality of the selected studies with the established quality index [53] and included 10 RCTs. Among the studies included in this systematic review, the methodological quality of studies was generally modest (mean score = 18.69, out of a highest possible score of 27). The quality index scale items with the highest scores in these studies were related to the reporting and external validity criteria. These items indicate strength in the clarity of the objective, main outcomes, participants’ characteristics, main findings, and the representatives of the participants to the whole general population. Scale items that were less satisfied, however, were those related to internal validity-confounding—particularly pertaining to allocation concealment and inadequate adjustment for confounding in the analyses. Blinding of participants and researchers tends to be difficult owing to the nature of interventions.

### Limitation

The findings of this review should be interpreted with consideration of some limitations. This review did not offer quantitative analyses (e.g., intention-to-treat and meta-analysis) on the effectiveness of low-intensity exercise due to the heterogeneity of the study designs. Five out of 15 studies were quasi-experimental studies in which participants were not randomly assigned to experimental or control groups; the efficacy of the quantitative analyses in yielding meaningful results may have thus been limited. Nevertheless, we included these studies in the review to enable a more comprehensive synthesis of the evidence. Although the quasi-experimental designs may have weakened the reliability of their study findings, it should be noted that the internal validity of the five quasi-experimental studies were scored as four [61, 64] to five [56, 57, 60], at moderate levels of quality (Table 1). High-quality RCT designs are strongly suggested for future research on the topic of low-intensity exercise and older adults to overcome this limitation. Another limitation of this study was the unavailability of sample size calculation, which may also affect the validity and reliability of the study findings. Future research endeavors would do well to include sample sizes and power calculations. Finally, a considerable portion of the included studies did not assess the intervention compliance rate, one of the potential key differences between exercises of different intensities [97–99]. It is therefore also suggested that future research provide such information.

## Conclusions

The findings from this review indicated that low-intensity exercise might offer both physical and cognitive health benefits to older adults aged 65 to 85 years—particularly among women, as shown in most of the included studies. Exercise could be of varying types, including but not limited to chair-sitting exercises, Tai Chi, walking, or stretching. In studies with high-risk populations (such as physically frail elders, nursing home residents, and fallers), low-intensity exercise intervention was useful in eliciting the desired physical and cognitive improvements. This finding is important, as most of the existing literature has focused on the benefits of moderate- and high-intensity exercise rather than those of low-intensity exercise. If low-intensity exercise is effective in promoting physical and cognitive health among older adults, it may be preferable when considering factors such as fall risk, safety, compliance, and effectiveness. Indeed, 7 of the included studies in the present review showed a satisfactory level of exercise intervention compliance rate for low-intensity exercise (>70 %) [53, 59, 64–68], whereas the remaining included studies did not report such information. It is, therefore, suggested that the exercise compliance of low-intensity exercise may be better than that of moderate- and high-intensity exercise, yielding better health benefits [97–99]. Further clinical application of low-intensity exercise still needs to be confirmed with additional research exploring best techniques and protocols for clinical populations. Incorporating cognitive training into low-intensity exercise programs (similar to Tai Chi) may also be a worthy research direction for future investigations.

## Appendix 1

### Quality Index [53]

Reporting: Were the following clearly described? (Y/N)

1. Study hypothesis/aim/objective

2. Main outcomes

3. Characteristics of the participants

4. Interventions of interest

5. Distributions of principal confounders in each group

6. Main findings

7. Estimates of random variability for main outcomes

8. All the important adverse events that may be a consequence of intervention

9. Characteristics of patients lost to follow-up

10. Actual probability values for main outcomes

External validity (Y/N/unable to determine)

11. Were participants who were asked to participate representative of the entire population from which they were recruited?

12. Were participants who were prepared to participate representative of the entire population from which they were recruited?

13. Were the staff, places, and facilities representative of the treatment the majority of participants received?

Internal validity – bias (Y/N/unable to determine)

14. Was an attempt made to blind participants to the intervention they received?

15. Was an attempt made to blind those measuring main outcomes of the intervention?

16. If any of the results of the study were based on “data dredging” was this made clear?

17. In trials and cohort studies, do analyses adjust for different lengths of follow-up? Or, in case-control studies, is the period between intervention and outcome the same for cases and controls?

18. Were appropriate statistical tests used to assess the main outcomes?

19. Was compliance with the intervention reliable?

20. Were main outcome measures reliable and valid?

Internal validity – confounding (selection bias) (Y/N/unable to determine)

21. For trials and cohort studies, were patients in different intervention groups? For case-control studies, were cases and controls recruited from the same population?

22. For trials and cohort studies, were participants in different intervention groups? For case-control studies, were cases and controls recruited over the same period of time?

23. Were participants randomized to intervention groups?

24. Was the randomized intervention assignment concealed from both patients and staff until recruitment was complete and irrevocable?

25. Was there adequate adjustment for confounding in the analyses from which main findings were drawn?

Power

27. Did the study have sufficient power to detect a clinically important effect where the probability for a difference due to chance was less than 5 %?
